# Repositioning of Antibiotics in the Treatment of Viral Infections

**DOI:** 10.1007/s00284-024-03948-7

**Published:** 2024-10-26

**Authors:** Anaíd Bustos-Hamdan, Jair Isidoro Bracho-Gallardo, Aída Hamdan-Partida, Jaime Bustos-Martínez

**Affiliations:** 1 Departamento de Atención a la Salud, Universidad Autónoma Metropolitana-Xochimilco, Mexico City, Mexico; 2 Maestria en Biología de la Reproducción Animal, Universidad Autónoma Metropolitana-Iztapalapa, Mexico City, Mexico

## Abstract

Drug repurposing, also known as drug repositioning, is a currently tested approach by which new uses are being assigned for already tested drugs. In this case there are antibiotics that are used to combat bacterial infections. However, antibiotics are among the drugs that have been studied for possible antiviral activities. Therefore, the aim of this work is to carry out a review of the studies of antibiotics that could be repositioned for the treatment of viral infections. Among the main antibiotics that have demonstrated antiviral activity are macrolides and glycopeptides. In addition, several antibiotics from the group of tetracyclines, fluoroquinolones, cephalosporins and aminoglycosides have also been studied for their antiviral activity. These antibiotics have demonstrated antiviral activity against both RNA and DNA viruses, including the recent pandemic virus SARS-CoV-2. Some of these antibiotics were selected in addition to its antiviral activity for their immunomodulatory and anti-inflammatory properties. Of the antibiotics that present antiviral activity, in many cases the mechanisms of action are not exactly known. The use of these antibiotics to combat viral infections remains controversial and is not generally accepted, since clinical trials are required to prove its effectiveness. Therefore, there is currently no antibiotic approved as antiviral therapy. Hence is necessary to present the studies carried out on antibiotics that can be repositioned in the future as antiviral drugs.

## Introduction

Antibiotics are a key element to combat infectious diseases caused by bacteria, and since their appearance have made it possible to reduce the morbidity and mortality associated with different pathologies in a very significant way [1]. Although each antibiotic has its own characteristics in terms of its pharmacology and spectrum of microbial action, it is necessary to take into account its classification to have a general view of its clinical usefulness. Within this classification are beta-lactams, glycopeptides, macrolides, lincosamides, aminoglycosides, quinolones, among others [2].

The treatment of viral infections has proven to be a challenge for health systems, since they are a serious threat to humans around the world, because there are many viral agents that cause infections. To this we must add that there is not a wide range of effective antivirals to combat and control viral infections. This has recently been exacerbated by the shortage of antiviral drugs and the recent appearance of emerging viruses such as severe acute respiratory syndrome coronavirus (SARS-CoV), Middle East respiratory syndrome coronavirus (MERS-CoV), Zika virus, the Chikungunya virus and the Ebola virus [3], and recently the appearance of SARS-CoV-2.

In general, research into efficient therapies against viruses has been much less successful than against bacteria. These differences may be due to the special characteristics of the life cycle of viruses, such as their intracellular replication, the use of cellular organelles, the high rates of replication and mutation, the possible integration into the host genome and the low number of antivirals existing. However, various groups of antibiotics have been tested for several years against different types of viruses that cause severe infectious diseases, as mentioned by Wang et al. [4], they found that teicoplanin, a semisynthetic glycopeptide used against Gram-positive bacteria, can inhibit the replication of pseudotyped Ebola viruses. They also found that it had no activity against three picornaviruses (naked viruses) and did not inhibit the pseudotyped Ebola virus after it was adsorbed to the cell surface. This suggests that teicoplanin inhibits virus entry.

The aforementioned work was one of the first studies carried out to reposition antibiotics for use in viral infections [4]. Drug repositioning is an approach that involves finding new uses for already approved drugs, which are used in various clinical treatments. This repositioning opens a new strategy in the treatment of viral infections. Therefore, research in bacteriology and virology that at first seems different may be able to be shared, as has been done in the case of antibiotics [5].

However, the limitations of drug repositioning must also be taken into account. Which implies: methodological challenges and legal problems, such as patents, which could prevent the entire process; another problem is the emergence of resistant microorganisms due to the use of an antibiotic for various diseases; Eliminate the concept of selectivity, open the possibility of one drug that can be used to treat two different diseases on its own. However, drug repositioning would contribute only a small part to solving the problem of an increasing number of new diseases and their treatment. That is why the objective of this work is a review of studies that demonstrate the antiviral activity of antibiotics and that could be repositioned for the treatment of viral infections. First, a brief description of the general characteristics of viruses will be made and then we will delve into the repositioning of antibiotics to fight viruses.

## General Characteristics of Viruses

According to metagenomic analyses, viruses are the most abundant microorganisms in the biosphere with an estimated 1031 types of viruses [6]. Viruses have peculiar properties that define them as intracellular infectious agents that depend on the cellular enzymatic machinery to replicate. They are characterized by having a very simple architecture that can protect a DNA or RNA genome in an envelope called a capsid (icosahedral, helical or complex) and that, in some cases, additionally has a lipid membrane. Progeny viral particles, called virions, are formed by self-assembly from components newly synthesized in the cell during its infectious cycle [6]. Some characteristics are equivalent to those of cells, such as the possession of genes, the ability to create multiple copies of themselves, and the ability to evolve by natural selection [7]. However, they do not perform autopoiesis, they cannot self-replicate because they do not have ribosomes or their own metabolism.

The classification system managed by the International Committee on Taxonomy of Viruses (ICTV) is based on the chemical nature of the nucleic acid of the viral genome (DNA or RNA, circular or linear, one or two strands, segmented or not), in the architecture and dimension of the capsid and in the presence or absence of a lipid membrane [6]. The ICTV also makes use of the Baltimore classification [8], which is based on the type of nucleic acid that the virus has and the way in which viruses produce their messenger RNA, which will be translated by the ribosomes of the host cells.

Viruses have different types of interaction with their respective hosts from a biochemical and cellular level to an ecological level in order to infect, replicate and spread. At the molecular and cellular level, there are specific determinants that allow a successful viral infection, such as binding to the host cell through viral surface proteins that bind to cellular receptors such as membrane proteins, lipids, carbohydrates, glycoproteins, polysaccharides, glycosphingolipids and lipopolysaccharides, among others. This binding determines the entry of the viral nucleic acid into the cell through its uptake and intracellular trafficking and, ultimately, penetration into the cytoplasm. This will cause, in some cases, such as viruses that infect vertebrates, to have cytopathic effects. In other cases, such as some viruses that infect vertebrates and some that infect prokaryotes, they can resort to a state of latency either through the integration of their genome into that of the cell or the formation of an episome [6].

The treatments that exist to destroy viruses are still few and are under investigation. However, the repositioning of drugs such as antibiotics has been one of the early strategies to treat viral infections, taking into account the understanding of their mechanisms of action and their possible effect on them. Therefore, it is possible to reuse drugs with an appropriate formulation to combat viruses safely and effectively [5].

## Repositioning of Antibiotics in Viral Infections

The repositioning or repurposing of drugs implies a change in their profile or therapeutic use. It is a way of finding new actions for existing proven drugs and is currently considered one of the main strategies to obtain treatments for emerging diseases or those that do not have treatments appropriate [9].

In recent years, bacterial or viral infections have been considered a serious public health problem, since they are responsible for almost a fifth of deaths worldwide [10]. Another problem that occurs today is the high resistance of microorganisms to drugs, which is why this has become a challenge for treating various infectious diseases. Therefore, the lack of effective treatments against antibiotic-resistant bacteria and recent viral epidemics represent a serious public health problem worldwide [11]. Antibiotics that have recently been approved by regulatory organisms have seen a decline in recent decades compared to previous decades. Due to this problem, this threat must be countered, it is necessary to look for strategies that help us mitigate this problem, the drug repositioning has emerged as an alternative procedure for the identification of effective drugs for the treatment of infectious diseases caused by viruses [11, 12]. Many investigations have positioned antibiotics as a promise in the fight against viral infections. A summary of the antibiotics and viruses against which antiviral activity has been demonstrated, with relevant references, is shown in Table 1. Also, a summary of relevant clinical trials conducted with antibiotics for the treatment of viral infections is shown in Table 1.

**Table 1:** Antibiotics with antiviral activity and clinical trials performed

Type of antibiotic	Antibiotic/antiparasitic	Virus on which it acts	Clinical trials^a^	References
Macrolides	Azithromycin	Rhinovirus	1 CT 0 ICTRP	[16]
		Respiratory syncytial virus (RSV)	4 CT 6 ICTRP [23, 25]	[21, 22]
		Influenza A	3 CT 0 ICTRP [26]	[17, 24]
		Zika	0 CT 0 ICTRP	[27–29]
		Hand-foot-mouth disease (HFMD)	0 CT 0 ICTRP	[30]
		SARS-CoV-2	122 CT 133 ICTRP [31, 35–38]	[18, 19, 24, 32]
	Clarithromycin	Rhinovirus	0 CT 0 ICTRP	[40]
		Influenza virus	1 CT 1 ICTRP	[43]
		SARS-CoV-2	3 CT 10 ICTRP [44–46]	[18, 19]
	Erythromycin	Zika	0 CT 0 ICTRP	[48]
		Dengue virus (DENV)	0 CT 0 ICTRP	[48]
		Yellow fever virus (YFV)	0 CT 0 ICTRP	[48]
		HCoV-OC43	0 CT 0 ICTRP	[49]
	Tulathromycin	Porcine reproductive and respiratory syndrome virus (PRRSV)	0 CT 0 ICTRP	[51]
	Ivermectin	Zika	0 CT 0 ICTRP	[52]
		Chikungunya	1 CT 1 ICTRP	
		Hendra	0 CT 0 ICTRP	
		YFV	0 CT 0 ICTRP	
		Sindbis	0 CT 0 ICTRP	
		Avian Influenza A	0 CT 0 ICTRP	
		West Nile	0 CT 0 ICTRP	
		Human Immunodeficiency Virus (HIV)	1 CT 1 ICTRP	
		SARS-CoV-2	91 CT 152 ICTRP [54– 56]	[53]
		BK polyomavirus (BKV)	0 CT 0 ICTRP	
		Equine Herpes simplex virus type 1	0 CT 0 ICTRP	
		Bovine herpes virus 1	0 CT 0 ICTRP	
		Pseudorabies	0 CT 0 ICTRP	
		Porcine circovirus 2	0 CT 0 ICTRP	
Glycopeptides	Teicoplanin	HIV	0 CT 0 ICTRP	[58–60]
		Influenza virus A and B	0 CT 0 ICTRP	[61]
		DENV	0 CT 0 ICTRP	[62]
		Hepatitis C virus (HCV)	0 CT 0 ICTRP	[63, 64]
		Ebola	0 CT 0 ICTRP	[4, 58]
		MERS-CoV	0 CT 0 ICTRP	[58]
		SARS-CoV-1	0 CT 0 ICTRP	[58]
		SARS-CoV-2	0 CT 1 ICTRP [70, 71]	[19, 58, 66, 67, 69]
	Vancomicyn	Feline coronavirus (FIPV)	0 CT 0 ICTRP	[58]
		SARS-CoV	0 CT 0 ICTRP	
		SARS-CoV-2	0 CT 0 ICTRP	
		HIV-1	0 CT 0 ICTRP	
		Influenza A and B	0 CT 0 ICTRP	[73]
		HSV 1 and 2	0 CT 0 ICTRP	
		Vaccinia	0 CT 0 ICTRP	
		YFV	0 CT 0 ICTRP	
		Zika	0 CT 0 ICTRP	
		RSV	0 CT 0 ICTRP	
		HCoV-229E	0 CT 0 ICTRP	
	Eremomicyn	FIPV	0 CT 0 ICTRP	[58, 72]
		SARS-CoV	0 CT 0 ICTRP	[58, 72]
		HIV-1 and 2	0 CT 0 ICTRP	[58, 72, 74]
		HCV	0 CT 0 ICTRP	[58, 72, 74]
	Ristocetin	Influenza A and B	0 CT 0 ICTRP	[75]
		HIV-1	0 CT 0 ICTRP	[58]
Tetracyclines	Doxycycline	DENV	0 CT 3 ICTRP [81]	[79, 80]
		Zika	0 CT 0 ICTRP	[82]
		Vesicular stomatitis virus (VSV)	0 CT 0 ICTRP	[83]
		SARS-CoV-2	7 CT 27 ICTRP [88–91]	[86, 87]
	Minocycline	Influenza	0 CT 0 ICTRP	[92]
		HIV	3 CT 3 ICTRP	[92, 94]
		Simian immunodeficiency virus (SIV)	0 CT 0 ICTRP	[93]
		West Nile	0 CT 0 ICTRP	[92, 95]
		Japanese encephalitis virus	0 CT 0 ICTRP	[92]
		DENV	0 CT 0 ICTRP	[92, 96]
		RSV	0 CT 0 ICTRP	[92, 97, 98]
	Eravacycline	SARS-CoV-2	0 CT 0 ICTRP	[99]
Fluoroquinolones	Ciprofloxacin	HCV	0 CT 0 ICTRP [102, 103]	[102, 103]
		BKV	0 CT 0 ICTRP [104]	[101–103]
		Vacuolated simian virus (SV40)	0 CT 0 ICTRP	[105]
		SARS-CoV-2	0 CT 0 ICTRP	[18, 19, 107, 108, 110–112]
	Moxifloxacin	BKV	0 CT 0 ICTRP	[101]
		SARS-CoV-2	0 CT 0 ICTRP	[18, 19, 107, 108, 110–112]
	Levofloxacin	BKV	1 CT 0 ICTRP	[101–103]
		HCV	0 CT 0 ICTRP	[102, 103]
		SV40	0 CT 0 ICTRP	[105]
		Influenza	0 CT 0 ICTRP	[106]
		SARS-CoV-2	0 CT 1 ICTRP	[18, 19, 107, 108]
Aminoglycosides	Gentamicin	HIV	0 CT 0 ICTRP	[117]
		SARS-CoV-2	0 CT 0 ICTRP	[118, 119]
Beta-Lactams	Cefuroxime	SARS-CoV-2	0 CT 0 ICTRP	[123]
	Azetidine-2	HCoV-229E	0 CT 0 ICTRP	[124]
		Influenza A and B	0 CT 0 ICTRP	
		Adenovirus 2	0 CT 0 ICTRP	
		Vaccinia	0 CT 0 ICTRP	
		Reovirus	0 CT 0 ICTRP	
		Parainfluenza 3	0 CT 0 ICTRP	
		Sindbis	0 CT 0 ICTRP	
		Coksakievirus B4	0 CT 0 ICTRP	
		YFV	0 CT 0 ICTRP	

^a^Number of clinical trial reports from the International Registry Clinical Trials Platform (IRCTP) [33] of WHO and Clinicaltrials.gov (CT) [34] of the National Institutes of Health of the USA, and relevant references of published clinical trials are also provided

### Macrolides (Azithromycin, Clarithromycin, Erythromycin, Tulathromycin, Ivermectin)

Macrolides are a type of broad-spectrum antibiotics with large molecular sizes, which include, among others: azithromycin (AZM), clarithromycin and erythromycin. Macrolides are usually well-tolerated, they are used to treat systemic and localized infections, which can occur in the eyes, skin, gastrointestinal tract, respiratory tract, and genital tract [13]. Regarding their mechanism of antibacterial action, macrolides inhibit protein synthesis. This occurs since these compounds bind reversibly to the 50S subunit of the bacterial ribosome, resulting in the inhibition of bacterial protein synthesis. The mechanisms that occur are: peptidyltransferase interference, the elongation of the polypeptide chain ends; inhibition of translocation; and premature detachment of peptidyl-tRNA from the ribosome [14]. Apart from their antibacterial action, several in vitro and in vivo studies in humans show results of the antiviral activity of macrolides in several virus families [15, 16].

Azithromycin has been one of the main antibiotics studied for its antiviral activity, this antibiotic has been considered broad-spectrum, has a long half-life and excellent tissue penetration [15, 17].

There are several studies of azithromycin and its activity against various types of viruses: rhinovirus, respiratory syncytial virus (RSV), influenza A, Zika, Ebola, enterovirus and coronaviruses including SARS-CoV-2, through various mechanisms [15, 17–19].

In most cases, pulmonary exacerbations in patients with cystic fibrosis are caused by respiratory viruses, especially human rhinoviruses, also these viruses can cause asthma exacerbations [20]. In these cases, according to Gielen et al. [16], they suggest that macrolides have antiviral and anti-inflammatory effects, in cell cultures, the antiviral properties of azithromycin were evaluated and it was shown that there was a seven-fold reduction in rhinovirus replication when azithromycin was used in bronchial cells compared to untreated cells. The action of azithromycin on bronchial cells was also found to be associated with several mechanisms that increase interferon (IFN) production, but the precise mechanism of action is unknown [16].

Azithromycin has also been shown to inhibit respiratory syncytial virus (RSV) replication, possibly by reducing the expression of the fusion protein receptor, the active A isoform of the Rashomologus (Rho) family, and subsequent inhibition of Rho kinase of human airway epithelial cells [21]. Azithromycin has also been shown to reduce the levels of IL-8 in nasal secretions caused by RSV, IL-8 is a dominant chemoattractant of neutrophils, which cause airway inflammation during RSV infection [22]. A clinical trial found that azithromycin may reduce morbidity in RSV-associated infections [23].

Influenza A virus infection is also inhibited by azithromycin, as reported by Du et al. [24], in lung cell lines. The mechanism of action is apparently the inhibition of the fusion of virus membranes and the cell vacuole, which occurs by increasing the pH of the vesicles containing the virus [17, 24]. In clinical trials with influenza patients, azithromycin has been shown to have anti-inflammatory effects [25, 26].

The Zika virus is a flavivirus transmitted by mosquitoes. This virus is associated with fetal anomalies and microcephaly, which are related to the damage of brain cells susceptible to this infection. Azithromycin reduces viral replication in glial cells and its activity is completed with daptomycin and sofosbuvir, which have anti-Zika activity [27]. Another reported finding is that azithromycin acts at a late stage of the viral cycle. It increases the expression of host type I and III interferons. It was also found that azithromycin positively regulates the expression of pathogen recognition receptors induced by Zika infection (MDA5 and RIG-I); increases phosphorylation of TANK-binding kinase 1 (TBK1) and interferon regulatory factor 3 (IRF3). These results demonstrate the possible use of azithromycin as an antiviral compound that can prevent the severe clinical problems associated with Zika, such as congenital microcephaly [28]. However, the exact mechanism by which azithromycin can inhibit Zika virus replication is not known [29].

Hand-foot-mouth disease (HFMD) is a viral disease that commonly occurs in infants; it is mainly caused by coxsackievirus A16 (CV-A16) and enterovirus A71 (EV-A71). The macrolides spiramycin (SPM) and azithromycin have been found to exhibit activity against CV-A16 and EV-A71, significantly reducing EV-A71 RNA and protein levels, possibly preventing viral RNA replication. It was found that EV-A71 variants resistant to spiramycin also presented similar resistance to azithromycin, indicating that the two drugs have a similar anti-EV-A71 mechanism. Animal experiments demonstrated that azithromycin has greater efficacy than spiramycin, greatly reducing disease symptoms and increasing survival in a mouse model infected with EV-A71. Therefore, it is suggested that azithromycin is an option in the treatment for HFMD induced by EV-A71, the safety of this drug has already been proven for infants and children, which makes its use in this disease even more promising [30].

In the current SARS-CoV-2 pandemic, one of the antibiotics used against COVID-19 was azithromycin. In the treatment of COVID-19, azithromycin was initially used alone or in combination with hydroxychloroquine [18, 19, 31, 32]. Its use was recommended in the early stages of the disease before complications developed. Azithromycin has been shown to block the entry of SARS-CoV-2 pseudovirus into several cell lines [24]. However, several clinical trials have been conducted or are ongoing to determine the use of azithromycin alone or with hydroxychloroquine for the treatment of COVID-19, there are currently 133 clinical trials reported in the International Registry Clinical Trials Platform (IRCTP) of WHO [33] and 122 in Clinicaltrials.gov (CT) of the National Institutes of Health of the USA [34], Table 1. Some clinical trials show encouraging results [31, 35], but others do not justify the use of azithromycin for the treatment of COVID-19 [36–38]. Due to these controversies the WHO did not recommend the use of azithromycin in the treatment of COVID-19.

**Fig. 1:**
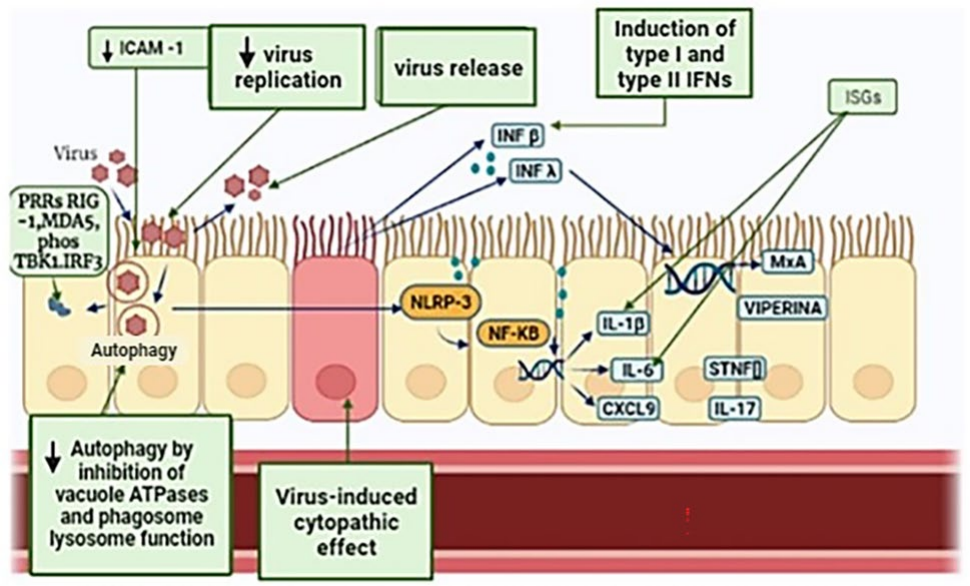
Antiviral effects of azithromycin (AZM). The main proposed mechanisms of the antiviral activities (green boxes) of azithromycin are shown. Inhibits virus replication and release. Also, inhibits autophagy and phagosome function. These actions are due it has an effect on type I and III interferon (IFNs), which cause a reduction in virus replication and release. It also increases the presentation of pattern recognition receptors (PRRs), induces the synthesis of viperin, myxoma resistance protein-1 (MxA) and interferon-stimulated gene (ISG). Upregulates viral pathogen recognition receptors melanoma differentiation-associated protein 5 (MDA5) and retinoic acid‐inducible gene I (RIG‐1). Increases levels of phosphorylated TANK-binding kinase 1 (phosTBK1), and interferon regulatory factor 3 (IRF3). Induces a reduction of IL‐6, IL‐8, IL‐17, C‐X‐C motif chemokine ligand 9 (CXCL9), tissue necrosis factor (TNF) and C-reactive protein (CRP). All these processes cause a possible action against some viral infections (Modified from Oliver and Hinks [15]) (Color figure online)

The exact mechanism by which azithromycin has activity against certain viruses is unknown. However, various mechanisms have been proposed. One of them proposes that azithromycin can inhibit endosome acidification during viral replication and infection, since the infection and replication of the virus requires acidification and endosomal cleavage processes. Azithromycin is a weak base and can accumulate in endosomal vesicles, making the pH more basic, thus inhibiting viral processes [17, 24]. Due to its immunomodulatory and anti-inflammatory effects, this antibiotic has been proposed as an alternative for patients with viral infections whose etiology involves the inflammatory response [18]. Since azithromycin is known to decrease the production of proinflammatory cytokines such as IL-6, IL-8, matrix metalloproteinases (MMP) and tumor necrotic factor alpha (TNF-α) [19]. In addition, it has been reported to reduce oxidative stress and modulate the functions of Th lymphocytes [19, 39]. Figure 1 shows the main mechanisms of action of azithromycin in viral infections.

Another macrolide that has had an effect on virus infection is clarithromycin on rhinovirus [40], which was found to suppress the production of IL-6, IL-1β, IL-8, and granulocyte and macrophage colony-stimulating factor [41]. It has also been seen to reduce the expression of the cell adhesion molecule (ICAM-1) gene in nasal fibroblasts [20]. ICAM-1 is known to be the receptor for most rhinoviruses, and the interleukins IL-6, IL-1β, and IL-8 play an important role in the pathophysiology of rhinovirus infection [42], so clarithromycin can modulate inflammatory processes during rhinovirus infection [40].

Clarithromycin has been used in avian influenza virus infections in monkeys [43]. Furthermore, it has been studied that clarithromycin in combination with chloroquine greatly improved the clinical status of a patient with COVID-19, and the RT-PCR against SARS-CoV-2 was negative in less than 14 days [18, 19]. There are few clinical trials so far on the use of clarithromycin in the treatment of COVID-19, giving encouraging results [44–46].

Like azithromycin, the exact mechanism for clarithromycin's antiviral activity is not known. However, clarithromycin has been observed to reduce the inflammation caused by infection and decrease vascular hyperpermeability by suppressing the induction of matrix metalloproteinases-9 (MMP-9) and monocyte chemoattractant protein-1 (MCP-1) [19, 47].

Erythromycin has also been shown to have activity in the treatment of infections caused by viruses. Such is the case of erythromycin estolate, which inhibits Zika virus infection, apparently blocking the entry of the virus, resulting in the virus being unable to infect cells, this effect has also been reported for other Flaviviruses such as dengue virus (DENV) and yellow fever virus (YFV) [48].

Erythromycin estolate has also shown activity against the coronavirus HCoV-OC43, it inhibits infection in different cell lines, apparently the mechanism of action is acting on the lipids of the viral membrane, damaging its integrity and releasing the viral RNA. Therefore, it acts in the early stages of the infection by inactivating the virus irreversibly at concentrations that do not produce a cytotoxic effect [49].

Another macrolide, tulathromycin, has also been studied in viral infections caused by porcine reproductive and respiratory syndrome virus (PRRSV). It was found that tulathromycin inhibits the necrosis produced by the virus, and also acts by inhibiting the inflammatory response induced by the virus in porcine macrophages [50]. Furthermore, tulathromycin has been used to increase the humoral response in pigs vaccinated against the swine influenza virus [51]. The mechanisms are not adequately understood.

Ivermectin deserves special mention, although it is not an antibiotic but an antiparasitic agent, it is also a macrolide derivative. Its antiviral activity has been demonstrated in several in vitro studies, where its activity against a large number of RNA viruses such as dengue, Zika, Chikungunya, Hendra, YFV, Sindbis, Avian Influenza A, West Nile, Human Immunodeficiency Virus (HIV), and SARS-CoV-2 is observed [52]. Ivermectin also has antiviral activity against DNA viruses such as BK polyomavirus, equine Herpes simplex virus (HSV) type 1, bovine herpes virus 1, pseudorabies, and porcine circovirus 2 [52]. The possible mechanisms of action of ivermectin are the extracellular inactivation of viruses, preventing the binding or entry of the virus, preventing the replication of the viral genome, preventing the synthesis of viral proteins and preventing the assembly or release of the virus [53].

Specifically, against SARS-CoV-2, ivermectin has been shown to prevent the entry of the virus, it also acts on important molecules of inflammation such as IFN, IL-6, and Toll-like receptors among others [53]. Multiple clinical trials have been carried out to see its efficacy against COVID-19, there are currently 152 clinical trials reported in the IRCTP and 91 in CT [33, 34]. Some reports with encouraging results [54] but many others that do not support its use in the treatment and prevention of COVID-19 [55, 56], Table 1. That is why the FDA and WHO issued statements not to use ivermectin for the treatment of COVID-19, so its use as an antiviral drug is still under study.

### Glycopeptides (Teicoplanin, Vancomycin, Eremomycin, Ristocetin)

Glycopeptides are a type of antibiotics that are responsible for inhibiting the transglycopeptidation and transpeptidation of the last stage of peptidoglycan biosynthesis of the bacterial cell wall. This group of antibiotics mainly includes vancomycin and teicoplanin [57]. Vancomycin is used to combat serious infections caused by multidrug-resistant Gram-positive pathogens. Several glycopeptides and its derivates, has also been shown to exhibit antiviral activities against various enveloped viruses such as: Coronaviruses, Influenza, HIV, Hepatitis C, Ebola, Flavivirus, HSV types 1 and 2, RSV, and Zika [4, 58], Table 1.

There are reports of teicoplanin and its derivatives, which inhibit viruses such as HIV, according to a study it is shown that teicoplanin interrupts the viral entry process [59, 60]. Likewise, an effect on the influenza virus has been found by lipophilic derivatives of teicoplanin pseudoaglycone, which presented a broad effect against influenza A and B viruses [61]. It has also been reported that teicoplanin exerts an in vitro action against dengue virus and other flaviviruses, interfering with the virus replication cycle in the early phase [62].

The use of teicoplanin has also shown inhibition of hepatitis C virus (HCV) replication; several teicoplanin derivatives present activity against this virus in infected cell cultures, with a new one being semi-synthetic derivatives of teicoplanin aglycone (LCTA-949), which showed efficiency in eliminating viral replicons from cells. When this compound was combined with HCV protease or polymerase inhibitors, an additive effect was observed [63]. A clinical study revealed that the use of teicoplanin improved a patient with hepatitis C [64].

Teicoplanin can inhibit the entry of Ebola, MERS-CoV and SARS-CoV viruses. Several investigations show that teicoplanin prevents the entry of the Ebola virus into cells by inhibiting the activity of cathepsin L. This same effect is observed with several glycopeptides derivatives: telavancin, oritavancin and dalbavancin [58, 65].

Regarding the possible action of teicoplanin against SARS-CoV-2, several studies have been conducted in this regard, firstly Yu et al. [66] determined the inhibition of pseudotyped virus of SARS-CoV-2 in cell lines, and later Bereckzi et al. [67] determined the inhibition of SARS-CoV-2 in Vero cells by perfluoroalkyl conjugates of teicoplanin, this apparently happens by preventing the cathepsin-mediated endosomal entry of SARS-CoV-2. Several studies have been conducted in this regard, suggesting that teicoplanin and its derivatives could be used as an alternative against SARS-CoV-2 infection [58, 68].

**Fig. 2:**
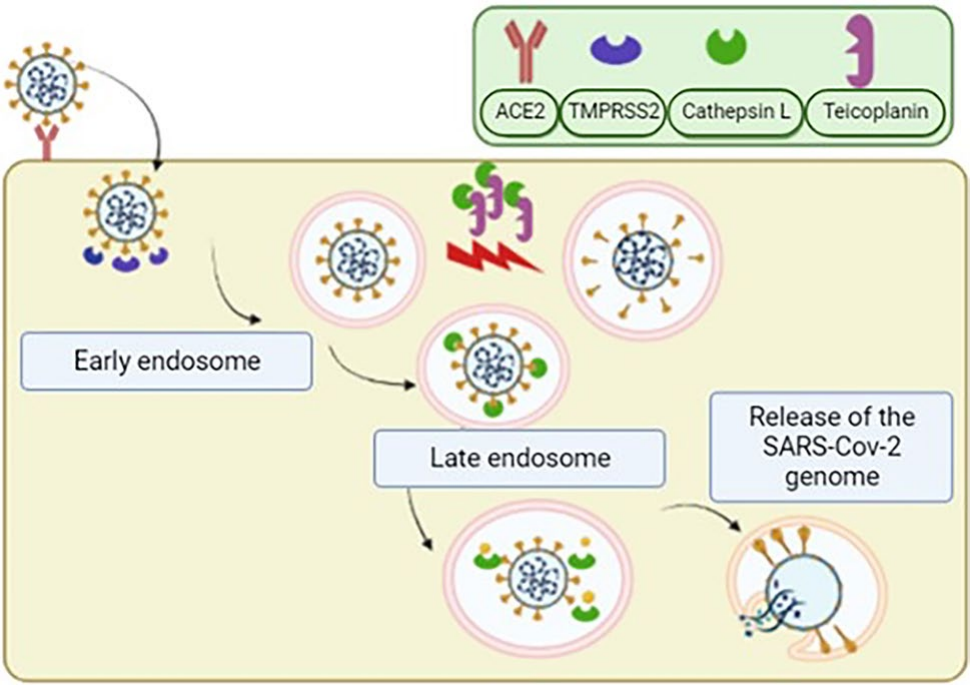
Scheme of teicoplanin that blocks the entry of SARS-CoV-2. The entry of SARS-CoV-2 into the target cells is observed. After binding of the virus to the cellular receptor angiotensin-converting enzyme 2 (ACE2), the proteolytic process is initiated by transmembrane serine protease 2 (TMPRSS2) on the cell membrane. Virions will be incorporated into endosomes, where protein S is further activated by cleavage with the cysteine protease cathepsin L. Cleavage of protein S by cathepsin L can be significantly blocked by teicoplanin (Modified from Baron et al. [47])

The precise mechanism by which teicoplanin exerts its antiviral activity has not been determined. However, it has been proposed that teicoplanin blocks the entry of SARS-CoV-2 by inhibiting the activity of cathepsin L (Fig. 2) [19, 66]. It has also been shown that teicoplanin derivatives can inhibit the entry of SARS-CoV-2 into cell lines since they inhibit the binding of the receptor binding domain (RBD) of the spike S protein with angiotensin-converting enzyme 2 (ACE2) [69]. On this basis, the use of teicoplanin has been recommended in both the prevention and treatment of people with SARS-CoV-2 infection [18, 19, 66]. Only two clinical trials have been published on the use of teicoplanin in the treatment of COVID-19, both showing positive results [70, 71]. However, the World Health Organization has not authorized the use of this or any other specific antibiotic to be used in SARS-CoV-2 infection in any clinical practice manual.

Vancomycin aglycone has activity in vitro against feline coronavirus (FIPV), and human coronaviruses SARS-CoV and SARS-CoV-2, and has also demonstrated activity against HIV-1 [58]. By introducing hydrophobic substituents to these vancomycin derivatives, compounds with greater antiviral activity are obtained [72]. Vancomycin aglycone hexapeptide derivatives were also evaluated in cell lines against RNA and DNA viruses, and were found to provide protection against influenza A and B viruses, HSV 1 and 2, vaccinia, YSV, Zika, RSV, and the human coronavirus HCoV-229E, so they could be considered broad-spectrum antivirals [73]. The possible mechanism of action is suggested to be inhibiting viral entry into the cell or inhibiting viral replication in early stages [58].

Eremomycin with hydrophobic substituents have enhanced activity against FIPV and SARS-CoV, likewise eremomycin aglycon with an (adamantyl-1) methyl substituent shows activity against HIV-1 and HIV-2 in cell lines [58, 72]. Furthermore, eremomycin aglycon derivatives have been shown to exhibit inhibitory activity against recombinant human protein kinases (PK), which are often important in viral infection, this inhibition may explain the antiviral activity of these compounds against unrelated viruses such as HIV and hepatitis C virus [74]

Ristocetin aglycon derivatives show anti-influenza in vitro activity [75], however, their clinical use is pending as they may cause platelet aggregation [76]. Other ristocetin aglycon derivatives conjugated with glucose, mannose or rhamnose also have activity against influenza A and B viruses apparently by preventing viral entry [75]. Ristocetin also shows activity against HIV-1 [58].

Two derivatives of teicoplanin pseudoaglycon, isoindole and benzoisoindole, also present activity against influenza virus and herpes virus [77].

### Tetracyclines (Doxycycline, Minocycline, Eravacycline)

Tetracyclines are broad-spectrum antibiotics with bacteriostatic activity, its lipophilic structure allows for high absorption in lung tissues. Their activity is due to the fact that they can interact with bacterial ribosomes by binding to the conserved region of the 16S rRNA, inhibiting the union of the aminoacyl-tRNA with the ribosome, which produces the inhibition of protein synthesis. Tetracyclines have high activity against several microorganisms: Gram-negative bacteria, Gram-positive bacteria, spirochetes, obligate intracellular bacteria and protozoan parasites [78]. In addition to this, it has been seen that tetracyclines also have antiviral activities [79].

The antiviral activity of tetracyclines was described by Sturtz [79], who studied the retroviral effect of doxycycline. He used various concentrations of doxycycline on a murine retrovirus-producing cell line. He found that the retroviral titer decreased significantly, the mechanism of this effect remains to be defined [79].

Doxycycline has also shown antiviral activity in vitro against the dengue virus, in a study the inhibition of the serine protease of the dengue virus (DENV2) by this antibiotic was demonstrated. The antiviral activity was tested in cell cultures, observing that the viral titer decreased significantly after adding doxycycline. The antibiotic broadly inhibited the entry and replication of four dengue serotypes [80]. A clinical trial of dengue patients treated with doxycycline showed a decrease in mortality [81].

Another study shows how doxycycline effectively inhibits the viral protease NS2B-NS3 of the Zika virus (ZIKV), which is important for virus replication, since this enzyme is responsible for breaking down the synthesized viral polyprotein, and releasing viral proteins that produce cytotoxic effects on ZIKV-infected cells. It was observed that viral replication was considerably reduced as the doxycycline concentration increased, also reducing cytopathic effects. It was found that the antiviral activity of doxycycline occurs by binding to the ZIKV protease inhibiting its catalytic activity. This study was carried out on human skin fibroblast, so treatment with doxycycline eliminated ZIKV infection and prevented cytopathic effects on the cells [82].

Another finding of the use of doxycycline is against the vesicular stomatitis virus (VSV), in a lung carcinoma cell line. Doxycycline has a high inhibition of viral replication, as well as the cytopathic effect produced by VSV. The action of doxycycline was observed in the early and middle stages of infection, this indicates that the drug does not prevent VSV entry into cells [83].

It is suggested that the antiviral action of doxycycline is a product of the regulation of the zinc-finger antiviral protein (ZAP), which prevents viral RNA from accumulating in the cytoplasm of the cell [84]. Among other characteristics of doxycycline as a senolytic drug is its ability to inhibit the replication of coronaviruses and be used to prevent pulmonary fibrosis [85]. Since in patients with viral infection it could exert an anti-inflammatory effect by inhibiting proinflammatory cytokines, such as IL-6 and TNF-α [81].

A docking study found that doxycycline is a possible candidate to treat COVID-19, as it can inhibit the main protease (Mpro) of SARS-CoV-2, also called 3-chymotrypsin-like protease (3CLpro) [86]. 3CLpro has a predominant role in the processing of viral proteins, mainly during the replication of RNA viruses, including SARS-CoV-2, and may be a possible target of action [18, 19, 87]. The potential use of doxycycline in the prevention and treatment of COVID-19 has been studied in several clinical trials, where it was found to reduce the risk of contracting the infection and help in treatment [88–90]. However, the PRINCIPLE trial developed in the UK found no improvement in patients with COVID-19 who received doxycycline, so they recommended not using it in treatment [91].

Minocycline is another tetracycline with a broad-spectrum of antiviral activity, since it has been seen to act against influenza virus, HIV, simian immunodeficiency virus (SIV), West Nile virus, Japanese encephalitis virus, dengue virus and RSV. It has been reported that minocycline reduces the damage caused by Japanese encephalitis virus in neuronal cell cultures in part by inhibiting oxidative stress, by preventing the synthesis of reactive oxygen species [92].

In the case of SIV, it was found that minocycline decreases the expression of the CD16 receptor of monocytes, therefore inhibiting the replication of SIV by preventing its entry into the cells, by inhibiting the passage of monocytes to the brain, it has a neuroprotective action [93].

With respect to HIV, it has been reported that minocycline decreases the activation of p38 and also inhibits virus replication in CD4+ T cells from primary cultures of human lymphocytes. Furthermore, minocycline has the ability to inhibit HIV reactivation in dormant CD4+ T lymphocytes, isolated from patients with clinically undetectable viremia. These effects possibly occur because minocycline reduces the expression of multiple cytokines that promote HIV replication and reactivation [94].

West Nile virus is capable of inducing caspase-dependent apoptosis in neuronal cells; minocycline inhibits both virus replication and its ability to induce apoptosis. The antiviral activity of minocycline is possibly due to the fact that it prevents the activation of c-Jun N-terminal kinase (JNK), which the virus requires for its replication [95].

In relation to the dengue virus, it has been reported that minocycline reduces the synthesis of viral RNA, also reducing the synthesis of virus proteins, therefore reducing the production of infectious virus. These effects were found for four serotypes of the virus of dengue in Hep-G2 cells. This is due to the fact that it reduces the phosphorylation of extracellular signal-regulated kinase (ERK), which is related to the pathogenesis and damage to organs by the dengue virus. Reducing ERK expression causes increased expression of antiviral genes including interferon α, 2′-5′-oligoadenylate synthetase 1 (OAS1), and 2′-5′-oligoadenylate synthetase 3 (OAS3). Therefore, the viral infection is reduced [96].

With respect to respiratory viruses, it has been reported that minocycline has activity against influenza virus and RSV. Minocycline can inhibit the replication of the H7N9 strain of influenza virus in Calu-3 cells [97]. In a study carried out in Hep-2 cells, it was shown that minocycline reduces the cytopathic effect of RSV and also prevents its infection. These effects are possibly due to affecting the production or maturation of the viral F protein, in addition to an increase in the cytokine CXCL10, which induces the production of interferon [98].

Finally, a computational study has shown that eravacycline, a synthetic antibiotic from the halogenated tetracycline class, is a good candidate against the main protease of SARS-CoV-2 [99].

### Fluoroquinolones (Ciprofloxacin, Moxifloxacin and Levofloxacin)

Fluroquinolones are a class of high-spectrum synthetic antibiotics. Fluroquinolones inhibit bacterial DNA gyrases, topoisomerase II and topoisomerase IV, which are necessary for DNA replication and transcription. Fluroquinolones have high activity against Gram-negative and positive bacteria, mycobacteria, and anaerobic bacteria [100]. Furthermore, it has been observed that these antibiotics have antiviral activity against DNA and RNA viruses [101].

In several studies it was found that ciprofloxacin, ofloxacin, levofloxacin and gatifloxacin can act in Hepatitis C virus (HCV) and BK polyomavirus (BKV) infections. When patients with HCV-induced chronic hepatitis were treated with ofloxacin, viral RNA decreased by at least one log titer [102]. However, when ciprofloxacin was used in patients with chronic Hepatitis C infection the effect appears to be limited [103].

In a study conducted in people with kidney transplants and persistent BK polyomavirus infection, it was found that after treatment with ciprofloxacin, the viral load was completely eliminated in three patients and decreased by more than 50% in three other patients [104]. In another study, the effectiveness of fluroquinolones in inhibiting the replication of the BK polyomavirus in vitro was demonstrated. Levofloxacin and ofloxacin inhibited BK polyomavirus replication in primary human kidney cells depending on the administered dose, producing a 77 to 90% inhibition in BKV load and significantly preventing cytotoxicity. Ciprofloxacin, levofloxacin, moxifloxacin, ofloxacin, norfloxacin, and gatifloxacin inhibited BK virus replication. The authors concluded that ofloxacin and levofloxacin inhibit, but do not completely prevent, BKV replication in human renal proximal tubule epithelial cells [101].

As previously stated, fluroquinolones are a powerful group of antibiotics that inhibit the helicase component of gyrase, inhibiting its activity, DNA tumor viruses also require helicases for DNA replication. In one study, the action of fluoroquinolones on the DNA replication of the vacuolated simian virus (SV40) was studied. To determine the ability to inhibit viral DNA replication, four different fluoroquinolones were studied: ciprofloxacin, levofloxacin, ofloxacin and trovafloxacin. The four quinolones produce inhibition of DNA replication and SV40 plaque formation in cultured cells. Furthermore, it was determined that each of these quinolones could inhibit the SV40 helicase. Therefore, fluoroquinolones and their derivatives could be used in the treatment of infections by human DNA viruses that act in the same way or are homologous to SV40 that depend on the helicase for their replication [105].

With respect to respiratory viruses, levofloxacin has also been used in the treatment of influenza virus infections since it produces benefits in the treatment of pneumonia caused by the influenza virus by inhibiting the response of inflammatory cells and suppressing the overproduction of nitric oxide in the lungs [106].

In the current SARS-CoV-2 pandemic, some studies have demonstrated the possible use of fluoroquinolones in the treatment of pneumonia associated with this virus and clinical trials with ciprofloxacin, levofloxacin and moxifloxacin have been suggested [18, 19, 107, 108].

A quinolone derivative, prodrome, used in the treatment of malaria has proven effective in patients with COVID-19, so this reinforces that fluoroquinolones could be used as antivirals in the treatment of SARS-CoV-2 infection [109].

Some of the mechanisms found are those exerted by ciprofloxacin and moxifloxacin, which can bind to the 3CLpro protease of SARS-CoV-2, inhibiting virus replication [110]. It has also been determined by docking that ciprofloxacin and norfloxacin-tetrazole hybrids could inhibit the main protease (Mpro) of SARS-CoV-2 [111]. Furthermore, fluoroquinolones may exhibit immunomodulatory activity, reducing the cytokine response, so they can be used to treat cytokine storm syndrome in COVID-19 [18, 19, 112].

Recently, nitroxoline, a hydroxyquinoline antibiotic, has been repositioned in the treatment of monkeypox virus since it inhibits virus replication in primary cell cultures by apparently inhibiting host cell signaling pathways [113].

### Aminoglycosides

Aminoglycosides have antibacterial activity, since they interfere with protein synthesis by binding to the aminoacyl site of the 30S subunit of the ribosome [114]. These drugs frequently present problems of nephrotoxicity and ototoxicity. Gentamicin, amikacin and tobramycin are the most commonly used aminoglycosides in the clinic. Aminoglycosides exhibit bactericidal activity against Gram-positive and negative bacteria and mycobacteria [115]. Furthermore, aminoglycosides have also been found to exhibit antiviral activities [116].

**Fig. 3:**
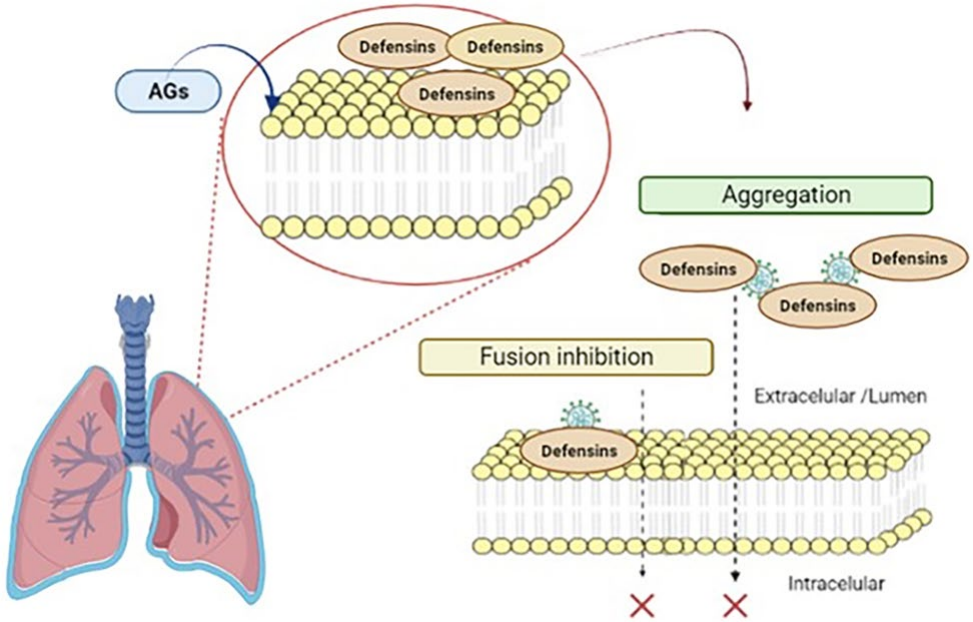
Possible mechanism of aminoglycosides in the inhibition of COVID-19 through defensins. Theta defensins exist as pseudo genes (they cannot be expressed) due to the presence of a premature termination codon. However, aminoglycosides (AGs) are believed to produce functional peptides from theta defensins (called retrocyclins) that inhibit virus fusion, suggesting that the addition of AGs to the COVID-19 therapeutic regimen could be beneficial

The study of aminoglycosides as antiviral treatment has been used for HIV, since these antibiotics interfere with the replication of the human immunodeficiency virus by disrupting essential RNA–protein contacts [117]. Another of the findings that have currently been had is its effect on SARS-CoV-2, which could be due to the production of retrocyclines, which are peptides produced from theta defensins, whose function is to inhibit fusion to cells and SARS-CoV-2 aggregation [118], Fig. 3. These defenses exert antiviral activity against enveloped or naked viruses [119].

However, SARS-CoV-2 infection in many cases produces a loss of smell, this aggravates the ototoxicity that is associated with the use of aminoglycosides. Therefore, the clinical use of these antibiotics in the treatment of patients with COVID-19 was rejected [120]. However, its potential use against SARS-CoV-2 remains.

### Beta-Lactams (Cefuroxime, Azetidine-2)

Cephalosporins are regularly used in elderly patients with community-acquired pneumonia, along with beta-lactamase inhibitors [121]. Cefuroxime is a broad-spectrum antibiotic, which is a second-generation cephalosporin. It is used to treat infections of the respiratory and genitourinary tracts, in addition to Lyme disease, and has good tolerability and safety profiles. Cefuroxime inhibits the third and final step of bacterial wall synthesis by binding to specific proteins in the bacterial wall called penicillin-binding proteins (PBPs) [122].

In a recent in silico study, the possible action of this antibiotic against three SARS-CoV-2 proteins was found: the main protease, RNA polymerase, and the protein S that forms the complex with ACE2 [123]. However, it is necessary to perform in vitro and in vivo tests to verify the properties found.

The azetidine-2-one ring is the core part of many antibiotics such as penicillins, cephalosporins, carbapenems and cephamycin, so it is named as azetidine-2-one (β-lactams). In a work carried out by Mandal et al. [124], they studied the antiviral activity of several phenyl azetidine-2-one sulphonyl derivatives, in Human embryonic lung cells (HEL), against the human Coronavirus 229E, Adenovirus-2 and Vaccinia virus. In green monkey Vero cells, they evaluated the action on Reovirus-1, Parainfluenza-3 virus, Sindbis virus, Coksakievirus B4, yellow fever virus and Punta Toro virus. They also studied the effect on a variant of the Influenza A virus H1N1 and another of the H3N2 type, in addition to a variant of the influenza B virus, on Madin-Darby Canine Kidney (MDCK) cells. In all cases they found a weak inhibition. The activity was the same for DNA or RNA viruses, so further study of the antiviral activity of these compounds is required [124].

## Discussion

In the studies reviewed we found that the repositioning of antibiotics is one of the strategies used for the treatment of viral infections. To expand these studies, the molecular mechanisms of replication, pathogenesis and virus-host interactions must be known; however, this is not known in several viruses and especially in emerging viruses [125].

One of the main antibiotics used in the repositioning with antiviral activity are macrolides and especially azithromycin, which has been shown to be safe, have good antibacterial activity and a long half-life. Various studies have shown that macrolides have antiviral properties in vitro against various viruses [15]. In several cases, AZM constantly emerges as a possible antiviral drug candidate against respiratory viruses and there are promising signs of its possible use in the clinic. Another property for its use in some viral infections is its anti-inflammatory activity, which may be important to reduce immunopathology, such as against pandemic beta-coronaviruses, where elevated inflammatory processes seem to be associated with mortality. Unfortunately, clinical trials conducted with azithromycin against COVID-19 so far show controversies where most of the trials conclude that there is no positive effect and therefore do not recommend its clinical use, which indicates that new well-designed clinical trials are required in patients with pandemic SARS-CoV-2 infections or new emerging viruses.

Glycopeptides are another type of antibiotic with a high probability of repositioning as antivirals due to the large number of viruses on which they can act in vitro. In the case of teicoplanin, as mentioned above, the mechanism that describes how it prevents viruses from entering cells is not known. However, to determine the molecular mechanisms involved in this process, should continue studying how this antibiotic inhibits the entry of Ebola and SARS-CoV viruses into cells [65]. To inhibit the entry of these viruses that require transport into the cell through endolysosomes to release their genetic material. Teicoplanin uses several host factors that are essential for the infection of the Ebola virus and SARS-CoV [99]. The use of teicoplanin in clinical practice is still being considered, although some articles mention that due to its low toxicity it could be used in the clinic, especially during emergencies that occur in outbreaks of serious viral infections [63]. So far, few clinical trials have been conducted with teicoplanin in the treatment of COVID-19, with positive results [70, 71], but they are not sufficient for the WHO to take it into account, so again more clinical trials are required to test the efficacy of this antibiotic in viral infections (Table 1).

In several cases the data found are the beginning of a possible use of antibiotics as antivirals, this represents another example of drug repositioning [126]. It is recommended that the results of these studies should confirm and precisely determine the concentrations that should be used within the therapeutic ranges [127], for the treatment of target viruses and have safe results that allow the use of antibiotics against viral infections. To date, no antibiotics have been approved by WHO as antiviral agents, but macrolides and glycopeptides are the most promising.

Furthermore, the use of antibiotics and their effect on viruses still need further studies, as most of the published research in vitro or by docking, both in vivo studies and clinical trials are needed to demonstrate the efficacy of antibiotics against viruses, so that health agencies including WHO allow the repositioning of antibiotics as antivirals.

## Conclusion

Currently the repositioning of antibiotics is one of the new strategies used in the treatment of viral diseases mainly against SARS-CoV-2. Although, as has been seen, promising results have been found in several antibiotics mainly macrolides and glycopeptides, their use as antiviral compounds remains controversial and is not widely accepted since clinical trials are required. Furthermore, the exact mode of action of the antiviral activity of most of these antibiotics is still not known. Therefore, it is necessary to continue studying antibiotics appropriately, including in vivo studies and clinical trials, so that with all the required evidence, repositioning them as antiviral agents becomes a reality. So, the idea that antibiotics only act against bacteria and not viruses could be changing.
